# Targeting Mechanosensitive Piezo1 Alleviated Renal Fibrosis Through p38MAPK-YAP Pathway

**DOI:** 10.3389/fcell.2021.741060

**Published:** 2021-11-05

**Authors:** Yuanyuan Fu, Pengzhi Wan, Jie Zhang, Xue Li, Jia Xing, Yu Zou, Kaiyue Wang, Hui Peng, Qizhuo Zhu, Liu Cao, Xiaoyue Zhai

**Affiliations:** ^1^ Department of Histology and Embryology, Basic Medical College, China Medical University, Shenyang, China; ^2^ Department of Nephrology, First Affiliated Hospital of China Medical University, Shenyang, China; ^3^ Department of Nephrology, Shengjing Hospital of China Medical University, Shenyang, China; ^4^ Institute of Nephropathology, China Medical University, Shenyang, China; ^5^ Department of Basic Medical College, China Medical University, Shenyang, China

**Keywords:** renal fibrosis, matrix stiffness, extracellular matrix secreation, Piezo1, YAP

## Abstract

Renal fibrosis is the most common pathological manifestation of a wide variety of chronic kidney disease. Increased extracellular matrix (ECM) secretion and enhanced microenvironment stiffening aggravate the progression of renal fibrosis. However, the related mechanisms remain unclear. Here, we evaluated the mechanism by which ECM stiffness aggravates renal fibrosis. In the present study, renal mesangial cells (MCs) were cultured on polyacrylamide hydrogels with different stiffness accurately detected by atomic force microscope (AFM), simulating the *in vivo* growth microenvironment of MCs in normal kidney and renal fibrosis. A series of *in vitro* knockdown and activation experiments were performed to establish the signaling pathway responsible for mechanics-induced MCs activation. In addition, an animal model of renal fibrosis was established in mice induced by unilateral ureteral obstruction (UUO). Lentiviral particles containing short hairpin RNA (sh RNA) targeting Piezo1 were used to explore the effect of Piezo1 knockdown on matrix stiffness-induced MCs activation and UUO-induced renal fibrosis. An *in vitro* experiment demonstrated that elevated ECM stiffness triggered the activation of Piezo1, which increased YAP nuclear translocation through the p38MAPK, and consequently led to increased ECM secretion. Furthermore, these consequences have been verified in the animal model of renal fibrosis induced by UUO and Piezo1 knockdown could alleviate UUO-induced fibrosis and improve renal function *in vivo*. Collectively, our results for the first time demonstrate enhanced matrix stiffness aggravates the progression of renal fibrosis through the Piezo1-p38MAPK-YAP pathway. Targeting mechanosensitive Piezo1 might be a potential therapeutic strategy for delaying the progression of renal fibrosis.

## Introduction

Chronic kidney disease (CKD) has become a serious public health problem endangering human health, which affects ∼10% of global population (Qiu et al., 2018). Renal fibrosis is the most common pathological manifestation of a wide variety of CKD, which characterized by excessive deposition of extracellular matrix (ECM) including collagen fibers and fibronectin (Imamura et al., 2018). Currently, there are very limited therapeutics to treat renal fibrosis effectively. So, the need to explore its unknown mechanisms and prevent the progression of this disease is urgent.

Mechanical information is increasingly recognized as comprehensive regulator of cell biological behavior. Abnormal mechanotransduction is correlated with the progression and severity of diseases including fibrosis (Li et al., 2019), cancer (Lee et al., 2019), and cardiovascular defects (Yamashiro et al., 2020). Increased ECM stiffness through collagen deposition or crosslinking accelerates ECM secretion and subsequently aggravates the progression of renal fibrosis, which forms a vicious positive feedback loop (Chen et al., 2014). Thus, targeting the mechanotransduction signaling pathway induced by enhanced ECM stiffness may provide a therapeutic strategy for renal fibrosis. As one of the inherent cells of the kidney, mesangial cells (MCs) have the function of maintaining the metabolic balance of ECM under normal physiological conditions (Huang et al., 2017). MCs can be activated and produce large amounts of ECM in renal fibrosis. However, the effect of extracellular matrix mechanical microenvironment on MCs is not clear.

The transcription factor Yes-associated protein (YAP) in the Hippo signaling pathway can be activated to translocated to nucleus by mechanical force stimulation (Dupont et al., 2011; Cobbaut et al., 2020). Furthermore, YAP binds to transcriptional enhanced associate domain (TEAD) in the nucleus, which affects target gene such as CTGF and then cell proliferation and differentiation, tissue regeneration and organ size determination (Zhang et al., 2014; Brusatin et al., 2018). It has been reported that increased ECM stiffness activates YAP, which leads to ECM deposition and then contributes to increased ECM stiffness (Cai et al., 2021). Despite these important findings, the molecular mechanisms by which MCs sense and transduce the mechanical signals to activate YAP needs to be further explored.

Piezo1, an emerging mechanical force receptor on the cell membrane, has been shown to respond to a wide range of mechanical forces, such as substrate stiffness, shear flow extrusion and tissue compression (Chen et al., 2018). Piezo1 can directly respond to mechanical force stimulation without the activation of other proteins or second messenger signals (Syeda et al., 2016). Reports have shown that Piezo1 is widely expressed in the kidney, bladder and endothelial cells (Dalghi et al., 2019). Piezo1-mediated sensing of mechanical force regulates physiological functions such as vasculogenesis (Li J. et al., 2014), bone homeostasis and innate immunity (Solis et al., 2019). However, it is not clear whether Piezo1 acts as an upstream mechanical response to YAP in renal fibrosis.

In our study, MCs were cultured on hydrogels with different stiffness *in vitro* to simulate the growth microenvironment of MCs in normal kidney and renal fibrosis. A series of *in vitro* knockdown and reverse experiments were performed to establish the signaling pathway responsible for matrix stiffness-induced MC activation. The results demonstrated that Piezo1 function as a sensor of matrix stiffness and play a crucial role in YAP-mediated ECM accumulation, and the signaling transduction was relied on p38 MAPK phosphorylation. Furthermore, an animal model of renal fibrosis induced by unilateral ureteral obstruction (UUO) was used to observe the Piezo1-p38MAPK-YAP signaling pathway also plays a role *in vivo* and the intervention of Piezo1 can alleviate the progression of renal fibrosis. This study explores the mechanism of renal fibrosis in terms of mechanical force, to provide a better perspective for preventing the process of renal fibrosis.

## Materials and Methods

### Animals

Six-to eight-week-old male C57Bl/6 mice weighing 20–25 g were obtained from the Liaoning Changshen to the Ethics code of the World Medical Association for the animal care and were approved by the Medical Ethics Committee of China Medical University.

### Unilateral Ureteral Occlusion Model and *in vivo* shRNA Treatment

The UUO model was established as described in previous study (Hoel et al., 2021). Briefly, the flank was exposed via a midline incision in the under aseptic conditions, the left ureter was exposed and ligated. Piezo1 shRNA and its negative controls were injected once with a dose of 2*10^7^ TU in 0.1 ml saline solution via the tail vein at 3 days after UUO, and the kidney specimens and blood samples were collected on 14 days after UUO operation. The shRNA sequences were as follows: Piezo1 shRNA, *5′-GCA​TCT​TTC​TCA​GCC​ACT​ACT-3’*.

### Histopathologic Examination of Renal Tissue

Paraffin-embedded kidney tissues were cut into 4 μm thick sections and mounted on slides. After deparaffinization and rehydration, the paraffin sections were stained with Masson’s trichrome (Jiancheng) to assess the histopathologic changes in the kidneys according to the manufacturer’s instructions. Three different fields were selected from per section, and three sections per animal were evaluated to obtain a mean value. Three mice from each group were used to obtain an overall value for subsequent statistical analysis.

For immunohistochemistry, after deparaffinization and rehydration, the paraffin sections were boiled with EDTA (pH = 8.0) for 2 min and 40 s at high pressure. The sections were then washed with PBS and incubated with goat serum for approximately 30 min. The sections were then incubated with anti-Fibronectin (ab2413, Abcam, 1:1,000),anti-α-SMA (130,621, Absin, Shanghai, China, 1:100) at 4°C overnight. Next the tissue sections were washed and incubated with biotinylated goat anti-rabbit IgG at room temperature for 1 h. The results were visualized with diaminobenzidine (DAB) (1809270031, MXB-BIO, Fuzhou, China). Hematoxylin was used as a counterstain. Images were taken with a camera mounted on a Nikon microscope (Eclipse 90i, Nikon, Tokyo, Japan).

### Preparation of Hydrogels

First, a 0.25% LAP initiator solution (EFL, Suzhou, China) was prepared in a brown bottle to protect it from light, and then incubated in a 60°C water bath for 30 min to dissolve. The solution was shaken every 10 min. The dissolved LAP standard solution was filtered with a 0.22 µm sterile filter for further use. Second, 0.5 g of the GelMA hydrogel (EFL, Suzhou, China) was put into a centrifuge tube, and the standard LAP initiator solution was added at different doses. The hydrogel was dissolved in a water bath at 60°C away from light for 30 min and filtered with a sterile filter. The hydrogel solution was then immediately injected into a Petri dish, and irradiated with an ultraviolet light source at 405 nm for approximately 1 min to solidify it.

### Substrate Stiffness Measurements

Atomic force microscope (AFM) was used to determine the stiffness and surface topography of the hydrogels. AFM experiments were conducted using a Bioscope Catalyst AFM (Dimension icon, Bruker Corporation, United States) set on an inverted fluorescence microscope (Nikon, Tokyo, Japan). The probe was an MLCT-type, and the cantilever had a nominal spring constant of 0.01 N/m. First, the AFM tip was instructed to contact the base of the Petri dish to obtain the force curves and then calibrate the deflection sensitivity of the cantilever. Second the spring constants of the cantilever beam were calibrated by the thermal tuning method. Subsequently, the AFM tip was controlled and made to contact the surface of the hydrogel on the Petri dish. AFM senses the deformation of a cantilever, the tip of which interacts with the sample surface, to measure the force between the tip and sample surface **(** Supplementary Figure S1A **)**. The morphology of the samples was measured by keeping the interaction between the tip and the surface constant (Li et al., 2014c). By pressing the tip onto the surface and measuring deformation of the cantilever, the stiffness of the samples was calculated using physical models (Liu et al., 2015). Fifty positions of the hydrogel were randomly selected from each group and used to obtain the force curves. Finally, the hydrogel young’s modulus was computed using the Hertz model (Li et al., 2014b). The roughness of hydrogel surface was flat relative to the cells **(** Supplementary Figure S1B **)**. The stiffness of the hydrogels was 2 ± 0.32 kPa and 50 ± 1.57 kPa, which are consistent with the elasticities of the normal kidney and sclerotic kidney, respectively **(** Supplementary Figure 1C **)**.

### Cellular Culture and Treatment

Immortalized mouse renal MCs (SV40-MES-13) were purchased from the Procell Life Science&Technology Co.,Ltd. (Wuhan, China) and cultured in DMEM/F12 containing 5% fetal bovine serum (FBS), 0.5% penicillin and streptomycin in an atmosphere of 5% CO_2_ at 37 °C. Following overnight serum deprivation, the SV40-MES-13 cells were seeded on cover slips coated with either 2 kPa (soft) or 50 kPa (stiff) hydrogels, and then treated with the YAP inhibitor Super-TDU (Selleckchem, Houston,TX, United States, s8554), Piezo1 activator Yoda-1 (Selleckchem, s6678), MEK1/2 inhibitor PD0325901 (MedChemExpress, Shanghai, China, HY-131295), JNK inhibitor SP600125 (MedChemExpress, HY-12041), or the p38 kinase inhibitor SB203580 (MedChemExpress, HY-10256) for 48 h.

### Lentiviral Short Hairpin RNA Transfection

Lentiviral particles containing short hairpin RNA (sh RNA) targeting Piezo1 and non-silencing shRNA were purchased from SyngenTech Co., Ltd. (Beijing, China). SV40-MES-13 cells were transfected with lentivirus containing Piezo1 shRNA to knockdown Piezo1, while the scrambled non-silencing shRNA sequence was used as a negative control (NC). The shRNA sequences were as follows: NC shRNA, *5′-*CCT​AAG​GTT​AAG​TCG​CCC​TCG​C*-3′*, Piezo1 shRNA, *5′-GCA​TCT​TTC​TCA​GCC​ACT​ACT-3’*. The transfection efficiency was determined by immunoblotting. Western blot analysis was used to verify the transfection efficiency **(** Supplementary Figure S2 **)**.

### Cell Viability Assay

Cell viability assay was performed with a Cell Counting Kit-8 (CCK-8) (MedChemExpress, Shanghai, China, HY-K0301) according to the manufacturer’s instructions. Briefly, cells were seeded in 96-well plates and treated with different concentrations of Super-TDU, the CCK-8 solutions were added and incubated for 4 h. Absorption at 450 nm was subsequently measured on the Varioskan Flash (Thermo Fisher, United States). Each experiment was repeated three times individually. The results were expressed as a percentage of the control that was set as 1.

### Real-Time PCR

Total RNA was isolated using TRIzol reagent (Invitrogen, Carlsbad, CA, United States) according to the manufacturer’s protocol. Briefly, 1 μg of RNA was reverse transcribed into cDNA using the Primer Script RT reagent kit (DRR047A, Takara, Japan). Quantitative PCR was carried out using an ABI 7500 real-time PCR system (Applied Biosystems, Darmstadt, Germany). The mRNA levels were normalized to GAPDH. The relative expression was calculated by the 2 (−ΔΔCt) method. The primer sequences are as follows: Piezo1 forward: 5′- TAC​GCC​GAG​GTG​TGC​TGG​AC -3′ and reverse: 5′- GCT​GGT​GTC​GTC​TGT​CAT​GCT​AC -3′.

### Western Blot Analysis

Renal tissues and SV40-MES-13 cells were lysed with RIPA lysis buffer containing protease and phosphatase inhibitors. The concentration of protein was estimated with a BCA protein assay kit (Beyotime Biotechnology, Shanghai, China), and the lysates were diluted until their protein concentration was to 2 μg/μl. After normalization, equal amounts proteins were separated on SDS-polyacrylamid gels. The samples were separated by applying a constant voltage of 100 V for 1.5 h and then transferred onto PVDF membranes. After nonspecific sites were blocked with Tris-buffered saline (TBS) containing 0.1% Tween 20 and 5% defatted dried milk, the membranes were incubated with anti-Piezo1 (78,446, Novas Biological, Littleton, Colorado, United States, 1:1,000), anti-p-p38MAPK (4,511, Cell Signaling Technology, 1:1,000), anti-p38MAPK (8,690, Cell Signaling Technology, 1:1,000), anti-*p*-ERK (4,370, Cell Signaling Technology, 1:1,000), anti-ERK (4,695, 1:1,000, Cell Signaling Technology), anti-*p*-JNK (sc6254, Santa Cruz Biotechnology, Dallas, TX, United States, 1:500), anti-JNK(sc7345, Santa Cruz Biotechnology, 1:500), anti-YAP (ab205270, Abcam, 1:1,000), anti-Collagen I (0,088, Wanleibio, Shenyang, China, 1:500), anti-Fibronectin (ab2413, Abcam, 1:1,000), anti-p-YAP (13,008, Cell Signaling Technology, 1:1,000), anti-MST1 (AF2367, Affinity Biosciences, 1:1,000), MST1 (22245-1-AP, Proteintech, 1:1,000), *p*-LATS1 (AF7170, Affinity Biosciences, 1:1,000), LATS1 (17049-1-AP, Proteintech, 1:1,000) and rabbit anti-GAPDH (7,021, Affinity, Shanghai, China, 1:1,000) at 4°C overnight. The blots were incubated with a horseradish peroxidase-conjugated goat anti-rabbit or anti-mouse secondary antibody (P448, DAKO, Glostrup, Denmark, 1:200) at room temperature for 1 h. Finally, the blots were visualized using a bioanalytical imaging system (Azure Biosystems, Dublin, CA, United States).

### Immunofluorescence Detection

Treated cells were fixed with 4% paraformaldehyde, washed with immunofluorescence detergent solution and blocked with 3% BSA for 30 min at room temperature. To visualize microfilaments, the cells were incubated with FITC-conjugated phalloidin (Beyotime Biotechnology, Shanghai, China) for 1 h at room temperature. To visualize YAP and α-SMA, the cells were incubated with anti-YAP (14,074, Cell Signaling Technology, 1:100), anti-α-SMA (130,621, Absin, Shanghai, China, 1:100) overnight at 4°C. Next day, the cells were washed and incubated with secondary antibody conjugated to the fluorescent markers FITC or TRITC at room temperature for 1 h in the dark, and then stained with 4′,6′-diamidino-2-phenylindole (Beyotime, Shanghai, China) for 10 min to visualize nuclei. The results were observed under a confocal microscope (Eclipse C1, Nikon, Tokyo, Japan). YAP nuclearstaining was quantified by counting the YAP staining-positive cells and then normalizing to the total cell number in each image. Three different fields were selected from a cell-cultured coverslip, and three parallel experiments for each treatment were performed.

### Statistics

All analyses were performed using SPSS software, version 21.0 (SPSS Inc, Chicago, IL, United States), and all experiments were performed at least in triplicate. Data on the net optical density of the bands are presented as the means ± standard deviations (SDs), and one-way analysis of variance followed by the Bonferroni test was used to compare the two different treatment groups. The unpaired two-sided Student’s *t* test was used to analyze difference between two groups. Differences with a *p* value of < 0.05 were considered statistically significant.

## Results

### Extracellular Matrix Stiffness Activates MCs *via* YAP

**FIGURE 1:**
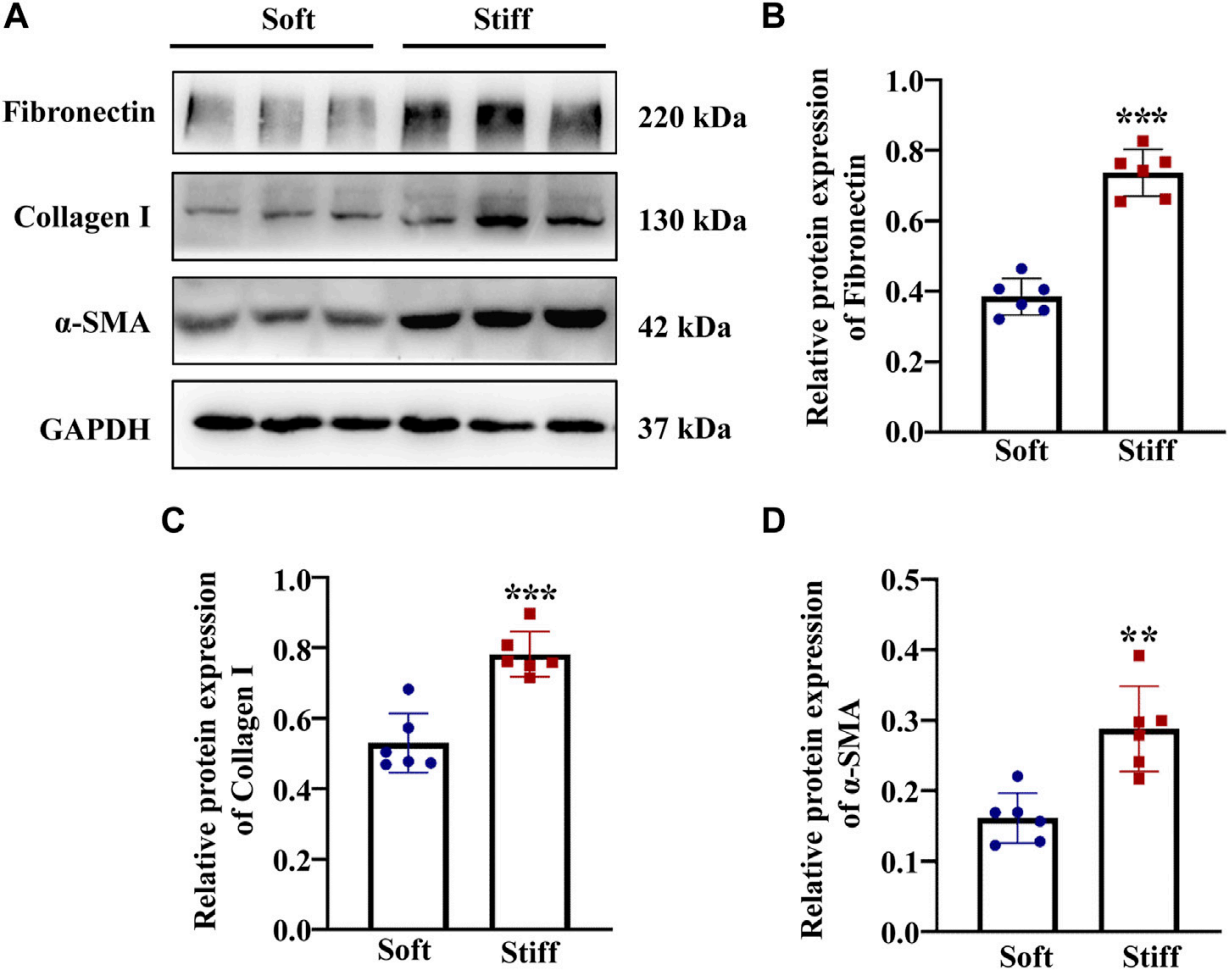
ECM stiffness activates MCs. MCs were cultured on hydrogels of different stiffness (2 kPa and 50 kPa) for 48 h. **(A)** Representative Western blot bands show expression levels of the myofibroblast marker α-SMA and ECM-associated proteins include of Collagen I and Fibronectin in cells cultured on soft and stiff hydrogels. The bar graphs show the results of semi-quantitative measurements of Fibronectin **(B)**, Collagen I **(C)** and α-SMA (D). (*n* = 6; *p < 0.05, **p < 0.01, ***p < 0.001).

**FIGURE 2:**
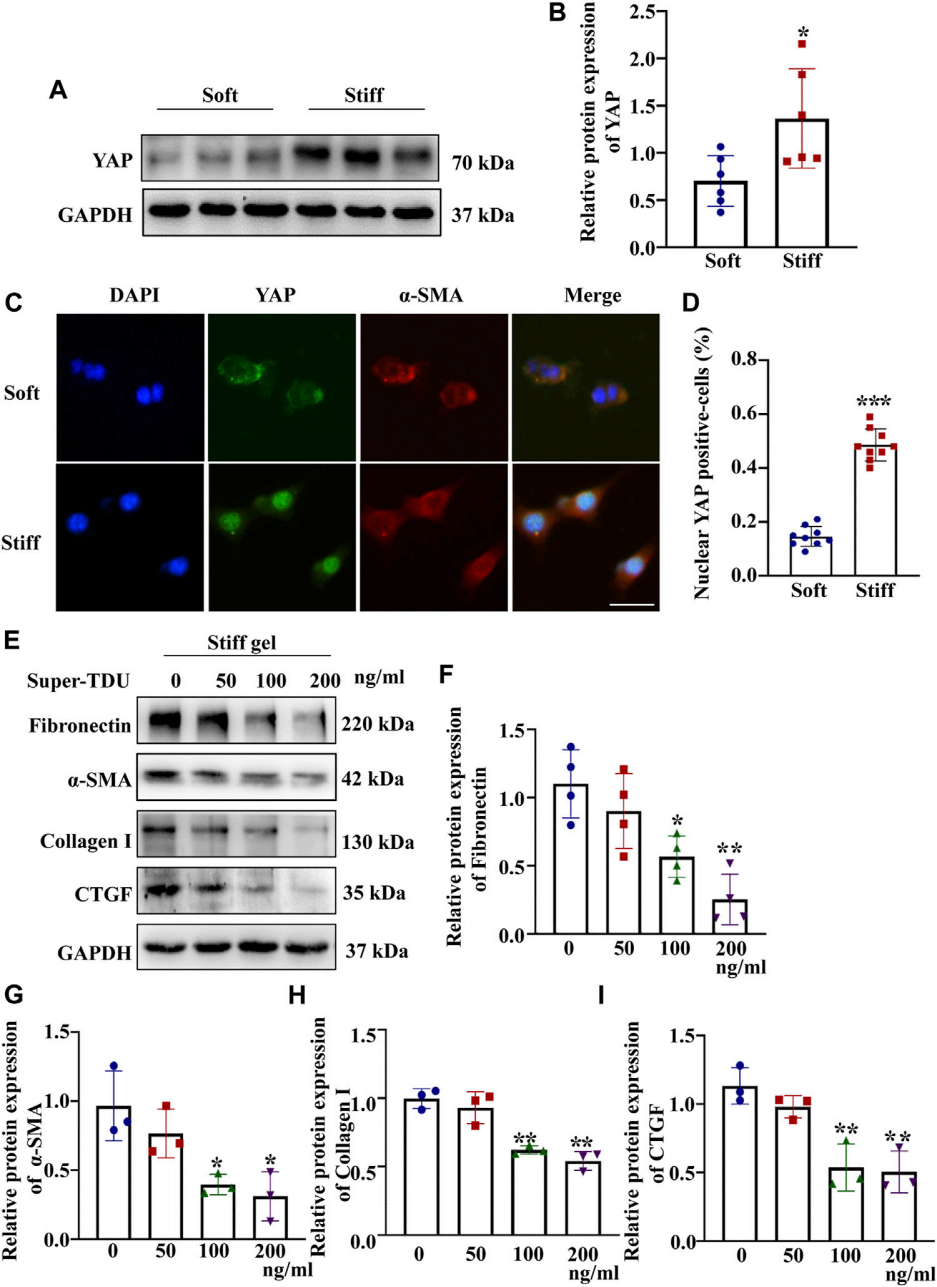
ECM stiffness activates MCs through YAP. MCs were cultured on hydrogels of different stiffness (2 and 50 kPa) for 48 h. **(A)** Representative Western blot bands show the protein expression level of YAP. **(B)** The bar graph shows the result of semi-quantitative measurement of YAP (*n* = 6; *p < 0.05, **p < 0.01, ***p < 0.001). **(C)** Representative photomicrographs show fluorescence staining with anti-α-SMA (red) and anti-YAP (green) antibodies in cells cultured on the soft and stiff hydrogels. Scale bar = 25 µm. **(D)** The percentage of cells with predominantly nuclear YAP staining. (*p* < 0.001). **(E)** Western blot bands show expression levels of Fibronectin, Collagen 1, α-SMA and CTGF after which MCs were incubated with Super-TDU at various concentrations on the stiff gel (50 kPa) for 24 h. The bar graph shows the results of semi-quantitative of Fibronectin **(F)**, α-SMA **(G)** Collagen 1 **(H)** and CTGF **(I)** measurements (*n* = 3; *p < 0.05, **p < 0.01, ***p < 0.001).

The mechanical properties of the cellular microenvironment play an essential role in determining cellular function and fate (Vining and Mooney, 2017). To test the effect of ECM stiffness on MCs, the protein expression of myofibroblast markers (α-SMA) and ECM-associated proteins (Collagen I and Fibronectin) was measured after the MCs had been cultured on matrices of different stiffness for 48 h. The results showed that the α-SMA, Collagen I and Fibronectin protein expression levels were higher in MCs cultured on the stiff (50 kPa) hydrogel than in those cultured on the soft hydrogel (2 kPa) (Figures 1B–D). Moreover, to test whether ECM stifness affects YAP in MCs, the expression and location of YAP were measured in cells cultured on the soft and stiff hydrogels. As shown in Figure 2B, YAP protein expression was significantly increased in cells cultured on the stiff hydrogel. In addition, YAP translocation into the nucleus from the cytoplasm increased as the stiffness of the hydrogel increased. Notably, cells cultured in hydrogel with greater stiffness showed localization of YAP to the nucleus in conjunction with the increased expression of the myofibroblast marker α-SMA (Figure 2C). In addition, pretreatment of cells with Super-TDU, a YAP inhibitor, specifically inhibited the expression of CTGF, Fibronectin, α-SMA and Collagen I in cells cultured on the stiffer hydrogel (Figures 2F–I). The dose of Super-TDU was selected on the basis of CCK-8 assay (Supplementary Figure S6), Based on these results, we selected 0, 50, 100, and 200 ng/ml as Super-TDU administration concentrations for subsequent experiments to eliminate the interference of Super-TDU-induced cell death. These results demonstrate that ECM stiffness activates YAP to promote MCs activation.

### p38MAPK Regulates Extracellular Matrix Stiffness-Induced YAP Activation

**FIGURE 3:**
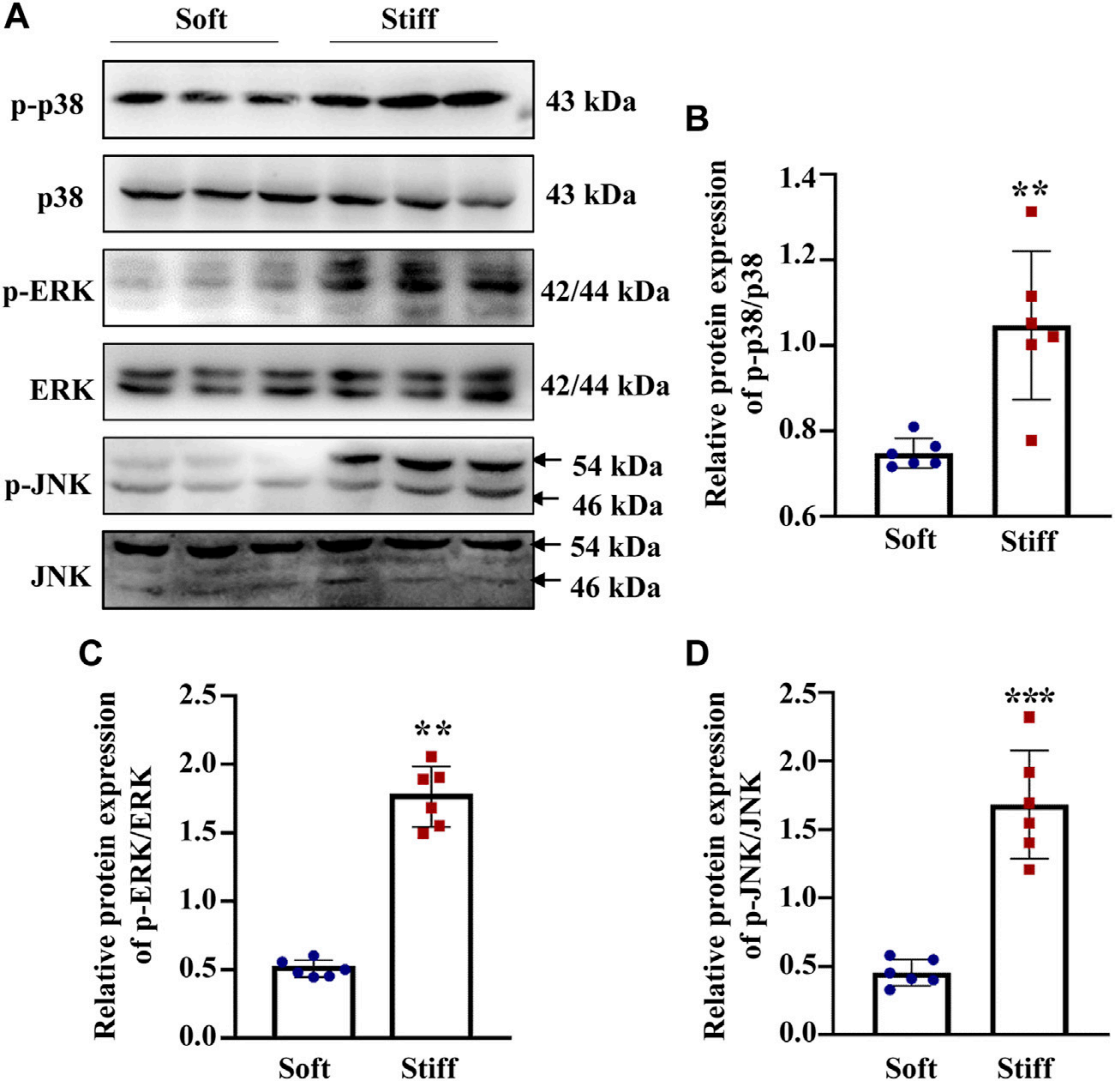
ECM stiffness promotes MAPK signaling pathway activation. **(A)** Representative western blot bands show the protein expression levels of p-p38, p38, *p*-ERK, ERK, *p*-JNK and JNK. **(B)**The bar graph shows the result of semi-quantitative measurement of p-p38/p38. **(C)** The bar graph shows the result of semi-quantitative measurement of *p*-ERK/ERK. **(D)** The bar graph shows the result of semi-quantitative measurement of *p*-JNK/JNK (*n* = 6; *p < 0.05, **p < 0.01, ***p < 0.001).

**FIGURE 4:**
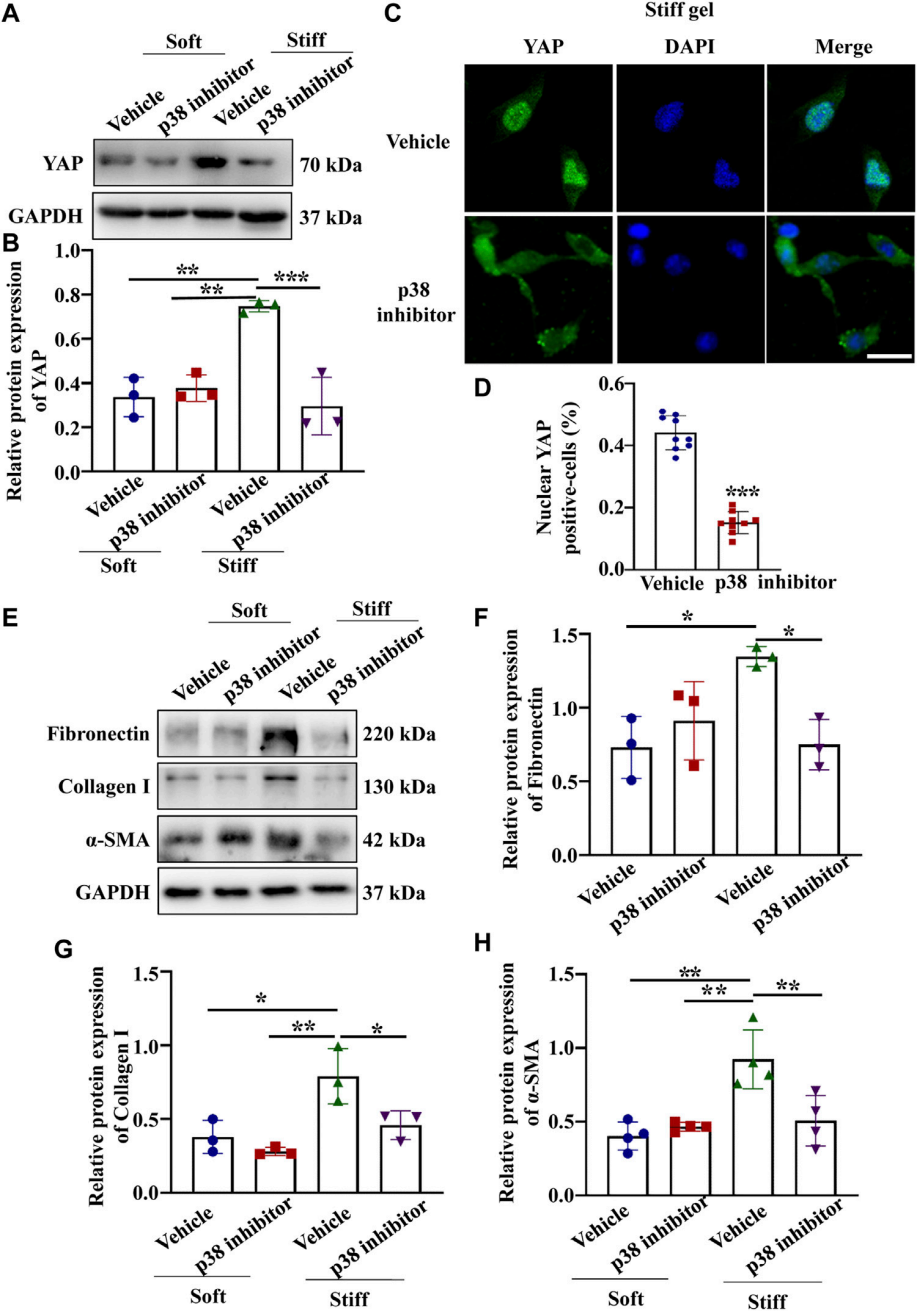
p38 inhibitor blocked YAP activation and ECM protein expression induced by enhanced hydrogel stiffness. MCs were treated with vehicle or a p38 inhibitor and cultured on hydrogels with different stiffness. **(A)** Representative Western blot bands showing YAP expression in each group. **(B)** Semi-quantitative measurement of YAP (*n* = 3; *p < 0.05, **p < 0.01, ***p < 0.001). **(C)** Representative photomicrograph showing fluorescence staining of YAP in cells treated with vehicle and a p38 inhibitor grown on the stiff hydrogels. Scale bar = 25 µm. **(D)** The percentage of cells with predominantly nuclear YAP staining. (***, *p* < 0.001). **(E)** Representative western blot bands show the protein expression levels of Fibronectin, Collagen 1 and α-SMA after MCs were treated with vehicle or a p38 inhibitor and cultured on the soft and stiff hydrogels. The bar graph shows the result of semi-quantitative measurement of Fibronectin **(F)**, Collagen I **(G)** and α-SMA(H) (*n* = 3; *p < 0.05, **p < 0.01, ***p < 0.001).

Having established that YAP plays a key role in mediating ECM stiffness-induced MC activation, we wanted to explore which signaling pathway regulates ECM stiffness-induced YAP nuclear localization. Studies have shown that the MAPK signaling pathway mediates the regulation of YAP activation. In our experiment, we found that cells cultured on the stiff hydrogel expressed more p-p38, *p*-JNK and *p*-ERK proteins (Figures 3B–D) than cells cultured on the soft hydrogel. To further evaluate the role of the MAPK signaling pathway in ECM stiffness-induced YAP activation, cells cultured on the stiffer hydrogel were treated with an MEK1/2 inhibitor (PD0325901), a JNK inhibitor (SP600125), or a p38 kinase inhibitor (SB203580). Interestingly, only SB203580, the p38 kinase inhibitor, inhibited YAP expression (Figure 4B) and blocked YAP nuclear localization (Figure 4D) induced by the enhanced stiffness. SP600125 and PD0325901 treatment did not alter the expression of YAP (Supplementary Figure S3). In addition, SB203580 decreased the expression of α-SMA, Collagen I and Fibronectin (Figures 4F–H). These results indicate that ECM stiffness induced YAP activation through p38MAPK.

### Piezo1 Mediates the Activation of YAP Through p38MAPK

**FIGURE 5:**
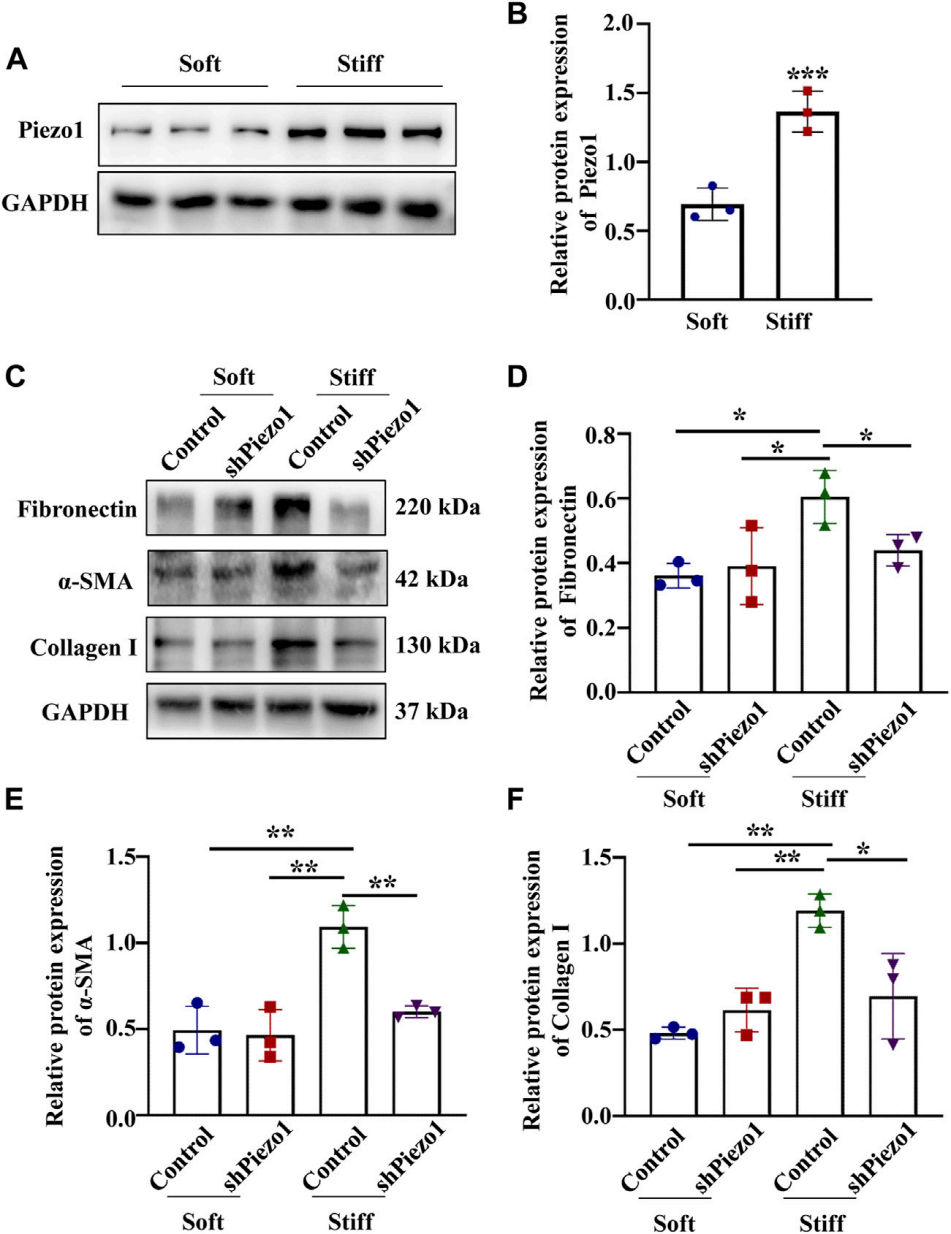
Piezo1 knockdown reduced ECM protein levels. **(A)** Representative western blot bands of Piezo1 in MCs cultured on hydrogels with a stiffness of 2 kPa or 50 kPa for 48 h. **(B)** The bar graphs show the results of semi-quantitative measurement of Piezo1 (*n* = 6; *p < 0.05, **p < 0.01, ***p < 0.001). **(C)** MCs were transfected with shNC or sh Piezo1 and grown on soft and stiff gels. Representative western blot bands of Fibronectin, α-SMA and Collagen I. The bar graph shows the result of semi-quantitative measurement of Fibronectin **(D)**, α-SMA **(E)** and Collagen I **(F)**. (*n* = 3; *p < 0.05, **p < 0.01, ***p < 0.001).

**FIGURE 6:**
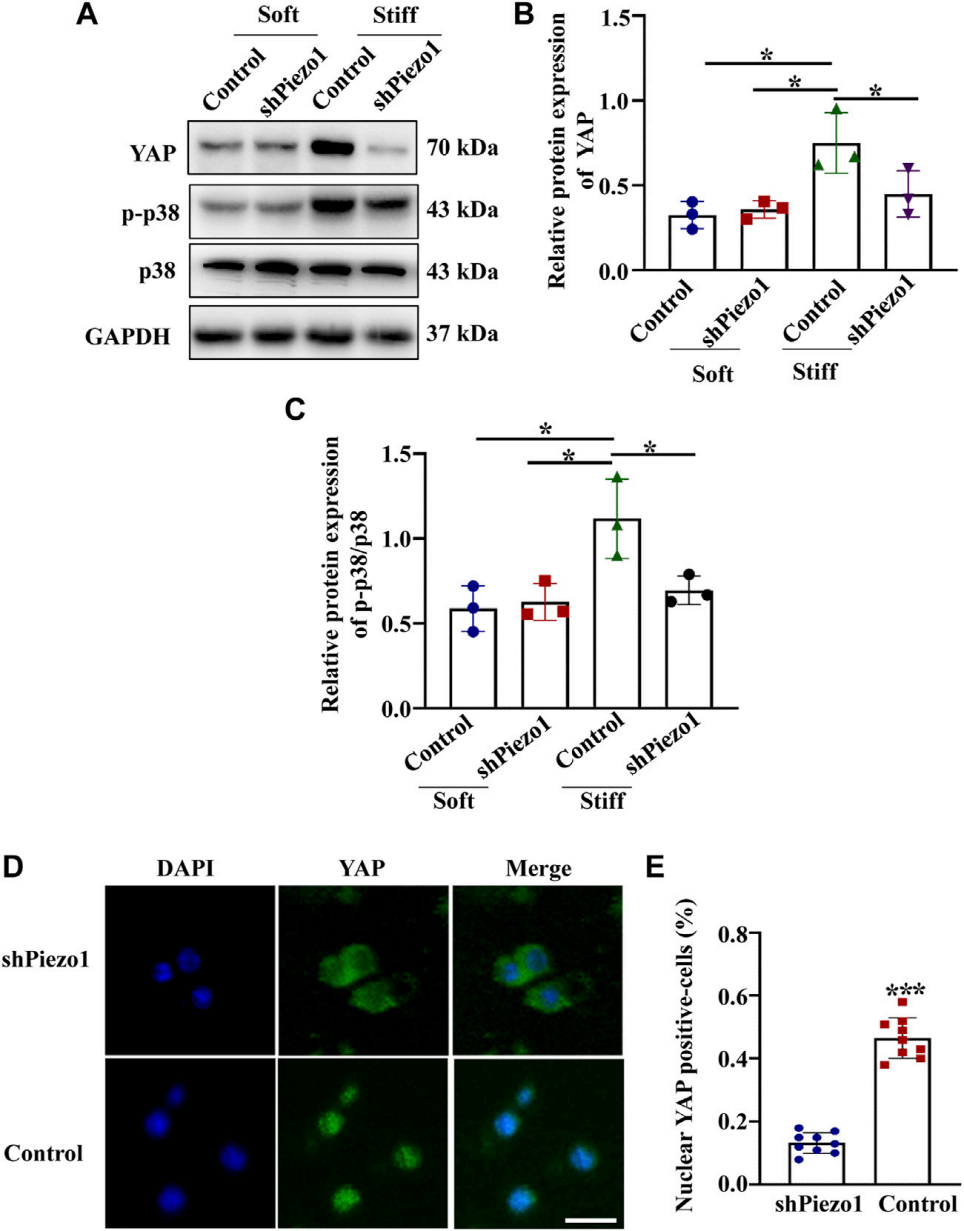
Piezo1 knockdown reduced YAP and p-p38 expression. MCs were transfected with shNC or sh Piezo1 and grown on soft and stiff gels. **(A)** Representative western blot bands of YAP, p-p38 and p38. Semi-quantitative measurement of YAP **(B)** and p-p38/p38 (*n* = 3 *p < 0.05, **p < 0.01, ***p < 0.001). **(D)** Representative photomicrographs show fluorescence staining with anti-YAP after MCs were transfected with shNC or sh Piezo1 and grown on stiff gels. Scale bar = 25 µm. **(E)** The percentage of cells with predominantly nuclear YAP staining. (*p* < 0.001).

Piezo1 can sense microenvironmental stiffness and transduce the external mechanical stimuli into the intracellular signaling pathway (Dasgupta and McCollum, 2019). In our experiments, we found that the Piezo1 protein expression level was increased in cells cultured on the stiff hydrogel compared to those cultured on the soft hydrogel (Figure 5B). To explore how Piezo1 mediates cellular responses to ECM stiffness, we silenced Piezo1 in MCs by transfection with lentiviral shRNA targeting Piezo1 and cultured the cells on stiff or soft hydrogel. Western blot analysis showed that Piezo1 knockdown reduced the protein levels of Fibronectin, α-SMA and Collagen I (Figures 5D–F). The Western blot results also showed that Piezo1 knockdown decreased p-p38 and YAP protein expression (Figures 6B,C). We also found that more cells transfected with Piezo1 shRNA displayed YAP nuclear exclusion than cells transfected with nontargeting shRNA (Figure 6E).

**FIGURE 7:**
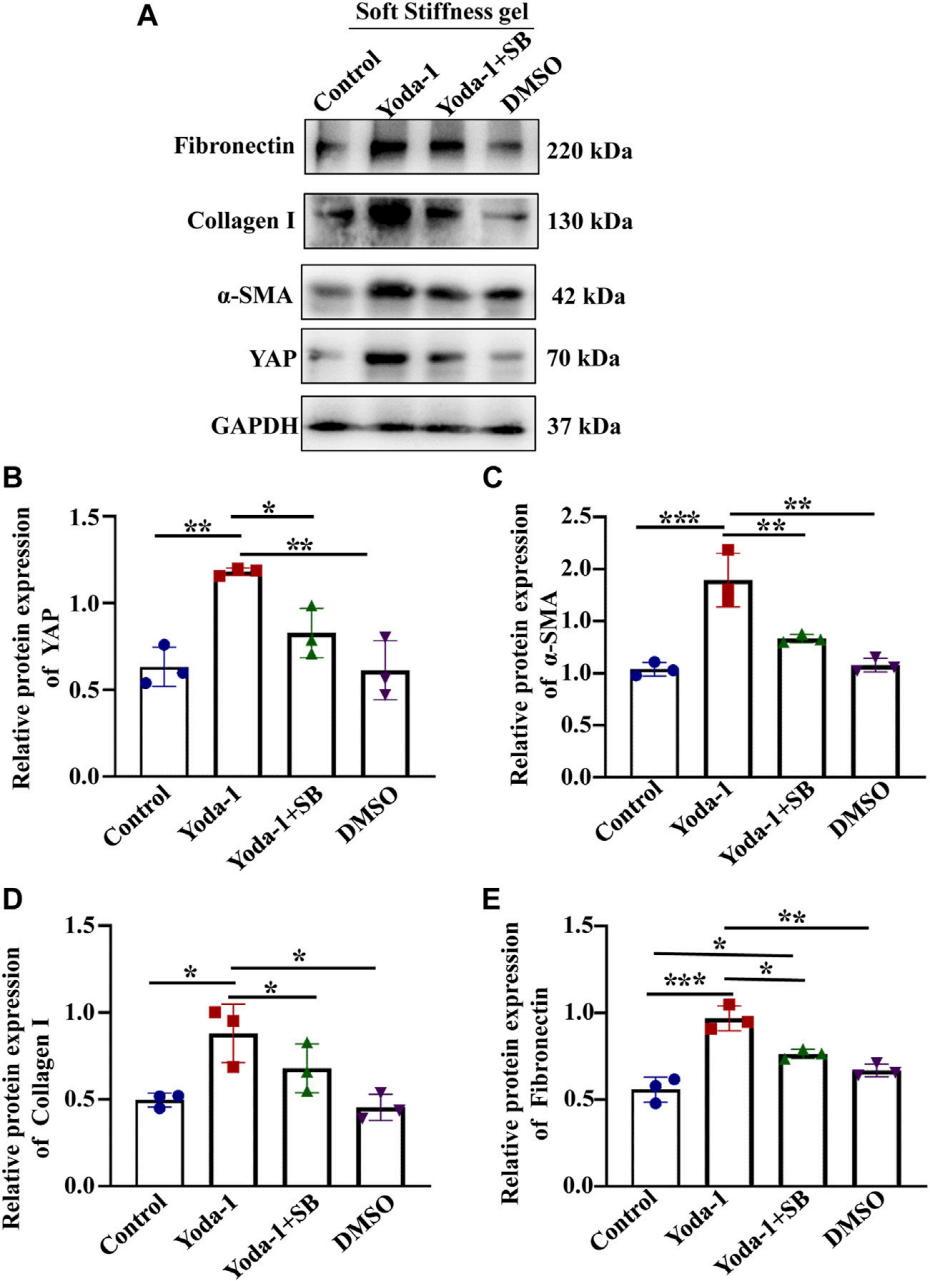
p38 MAPK inhibitor reduced the increased YAP expression and ECM secretion induced by Piezo1 activator. **(A)** MCs were incubated with Yoda-1 and SB for 48 h on the soft gel. **(A)** Representative western blot bands represent the protein expression levels of Fibronectin, Collagen 1, α-SMA and YAP. The bar graph shows the result of semi-quantitative measurement of Fibronectin **(B)**, α-SMA **(C)**, Collagen I **(D)** and YAP **(E)**. (*n* = 3; *p < 0.05, **p < 0.01, ***p < 0.001).

We then treated cells grown on the soft hydrogel with a p38 kinase inhibitor (SB203580) and a specific Piezo1 agonist (Yoda-1). we found that cells treated with Yoda-1 displayed higher YAP, Fibronectin Collagen I and α-SMA expression (Figures 7B–E) than the control cells. Moreover, the expression of YAP, Fibronectin Collagen I and α-SMA expression reduced in cells treated with Yoda-1 and SB203580 when compared with cells applied for Yoda-1 alone. These results suggested that Piezo1 induced nuclear YAP translocation through p38 MAPK as ECM stiffness increased.

Piezo1 Knockdown Alleviated Renal Fibrosis Induced by UUO through p38MAPK-YAP Pathway.

**FIGURE 8:**
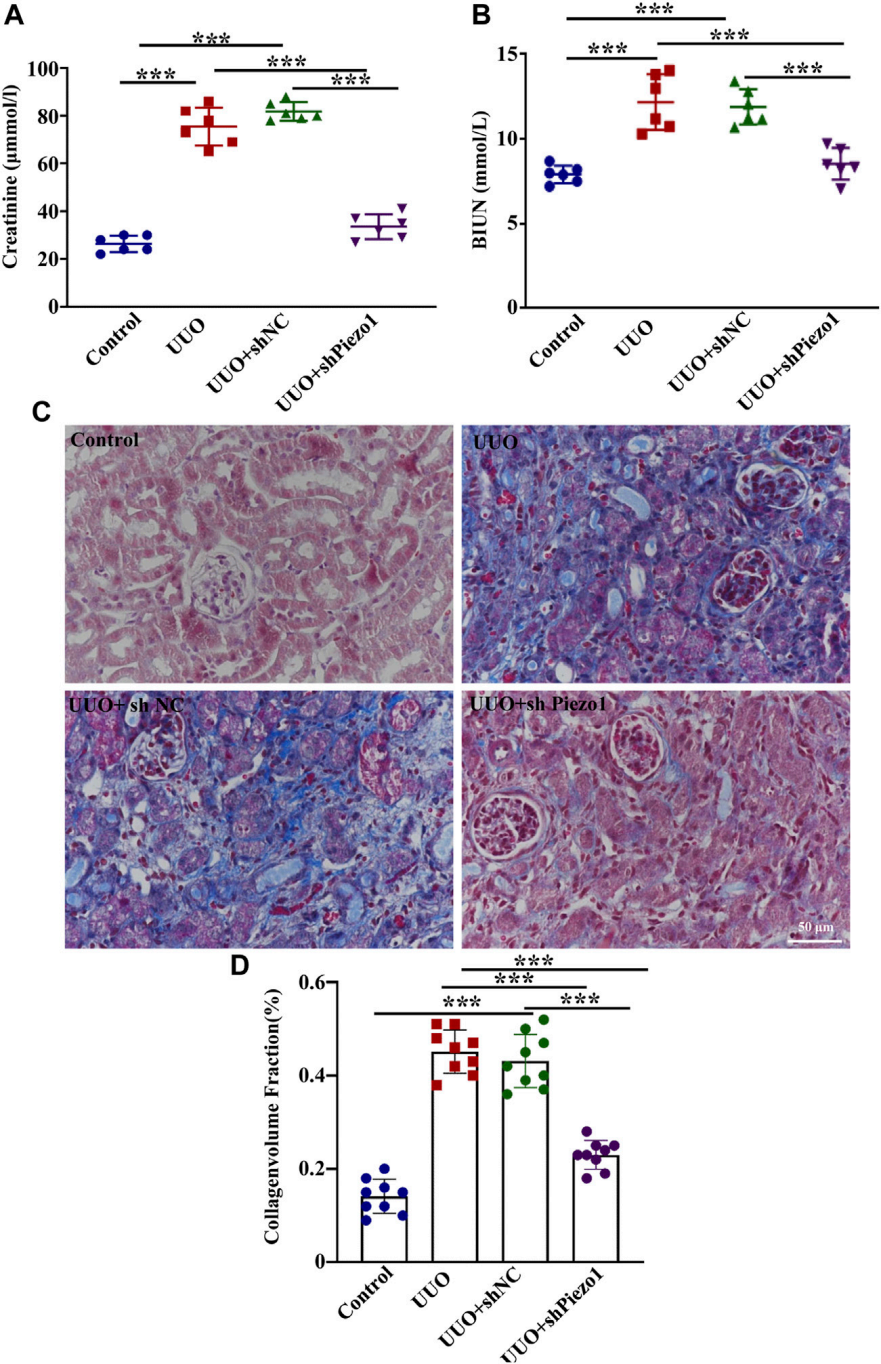
Piezo1 knockdown improved renal function in UUO mice. An animal model of renal fibrosis induced by UUO was established and a loss-of-function experiment was carried out by using shRNA of Piezo1 and shNC in UUO mice. shNC refers to empty vector without Piezo1 shRNA. Renal function is assessed by **(A)** plasma creatinine and **(B)** BUN levels. (*n* = 6; *p < 0.05, **p < 0.01, ***p < 0.001). **(C)** Masson staining reveals the excessive deposition of ECM (blue) in four different treatment groups. **(D)** The collagenvolume fraction in four different treatment groups (*p < 0.05, **p < 0.01, ***p < 0.001).

**FIGURE 9:**
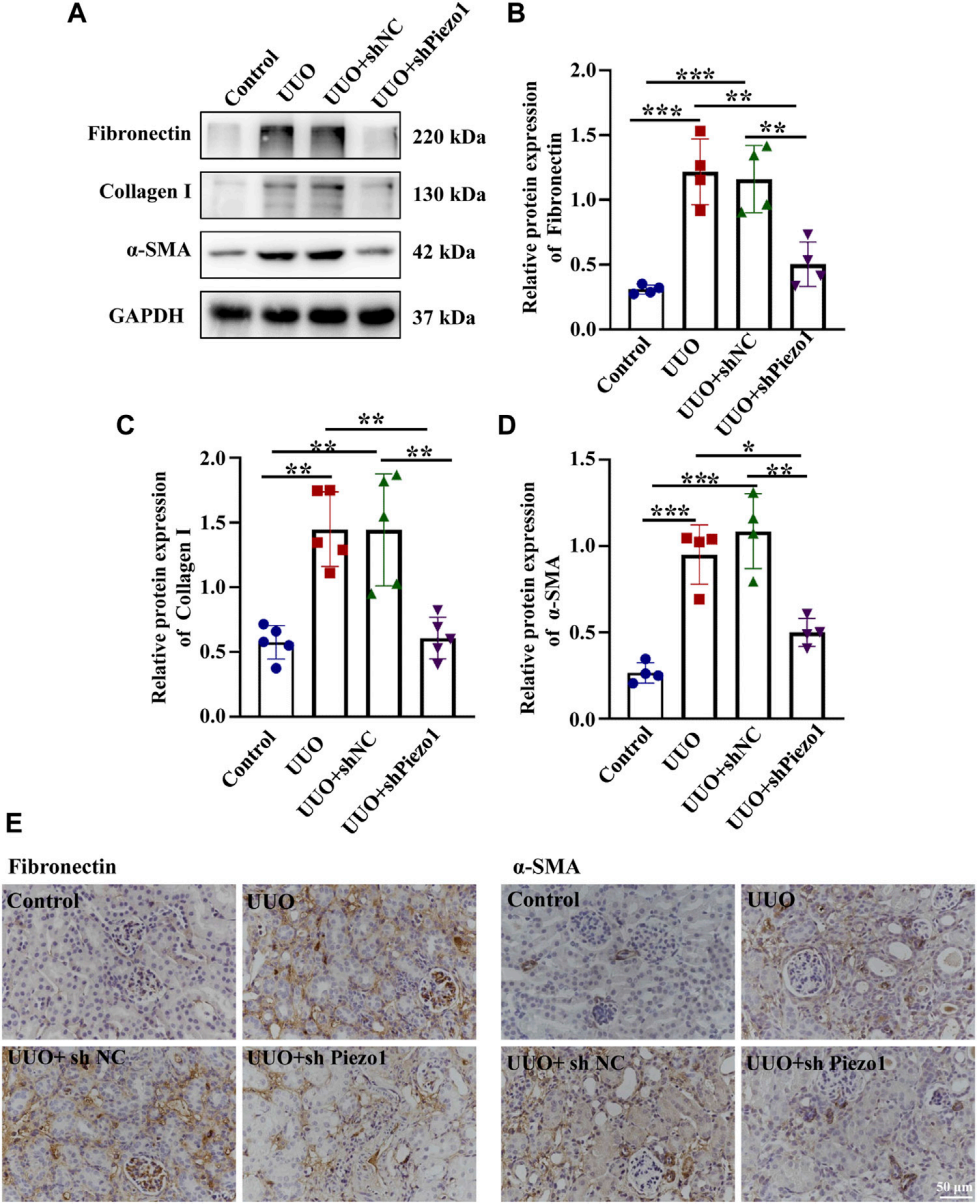
Piezo1 knockdown reduces the expression of ECM protein levels in UUO mice. An animal model of renal fibrosis induced by UUO was established and a loss-of-function experiment was carried out by using shRNA of Piezo1 and shNC in UUO mice. **(A)** Representative western blot bands show the protein expression of Fibronectin, Collagen I and α-SMA in the four different treatment groups. The bar graph shows the result of semi-quantitative measurement of Fibronectin **(B)**, Collagen I **(C)** and α-SMA **(D)**. (*n* = 4; *p < 0.05, **p < 0.01, ***p < 0.001). **(E)** Representative photomicrographs show immunohistochemical staining with Fibronectin and α-SMA after Piezo1 knockdown in UUO mice. scale bar indicates 50 μm.

To explore whether Piezo1-p38MAPK-YAP signaling pathway play a role in renal fibrosis and block the pathway could provide a potential therapeutic target for postponing the development of renal fibrosis, an animal model of renal fibrosis induced by UUO was established and a loss-of-function experiment was carried out by using shRNA of Piezo1 in UUO mice. Firstly, we found that the UUO group showed increased serum levels of BUN and creatinine as compared to the control group, whereas in Piezo1 knockdown UUO mice, this apparent upregulation was alleviated. The serum levels of BUN and creatinine did not differ between the UUO group and UUO + sh NC group (shNC refers to empty vector without Piezo1 shRNA) (Figures 8A,B). Masson staining further demonstrated that renal fibrosis was alleviated in Piezo1 knockdown UUO mice. The results showed that comparison with control group, the deposition of blue stained collagen fibers significantly up-regulated in the peritubular interstitial region in UUO and UUO + sh NC treated groups, however, the collagen fibers deposited were significantly reduced when Piezo1 was knocked down in UUO group (Figure 8D). In addition, compared to the UUO group, Piezo1 knockdown UUO mice showed decreased levels of the renal fibrosis markers α-SMA, Collagen I and Fibronectin (Figures 9B–D). Consistent with the results, immunostaining staining shown that α-SMA was widely expressed in the perivascular interstitial region and in smooth muscle cells in UUO and UUO + sh NC groups, and the α-SMA expression significantly reduced after Piezo1 shRNA administration, and was mainly expressed in smooth muscle cells. The fibronectin protein was widely expressed in the interstitial region in UUO and sh-NC treated UUO groups, whereas the distribution was significantly decreased in UUO treated with Piezo1 shRNA group (Figure 9E).

**FIGURE 10:**
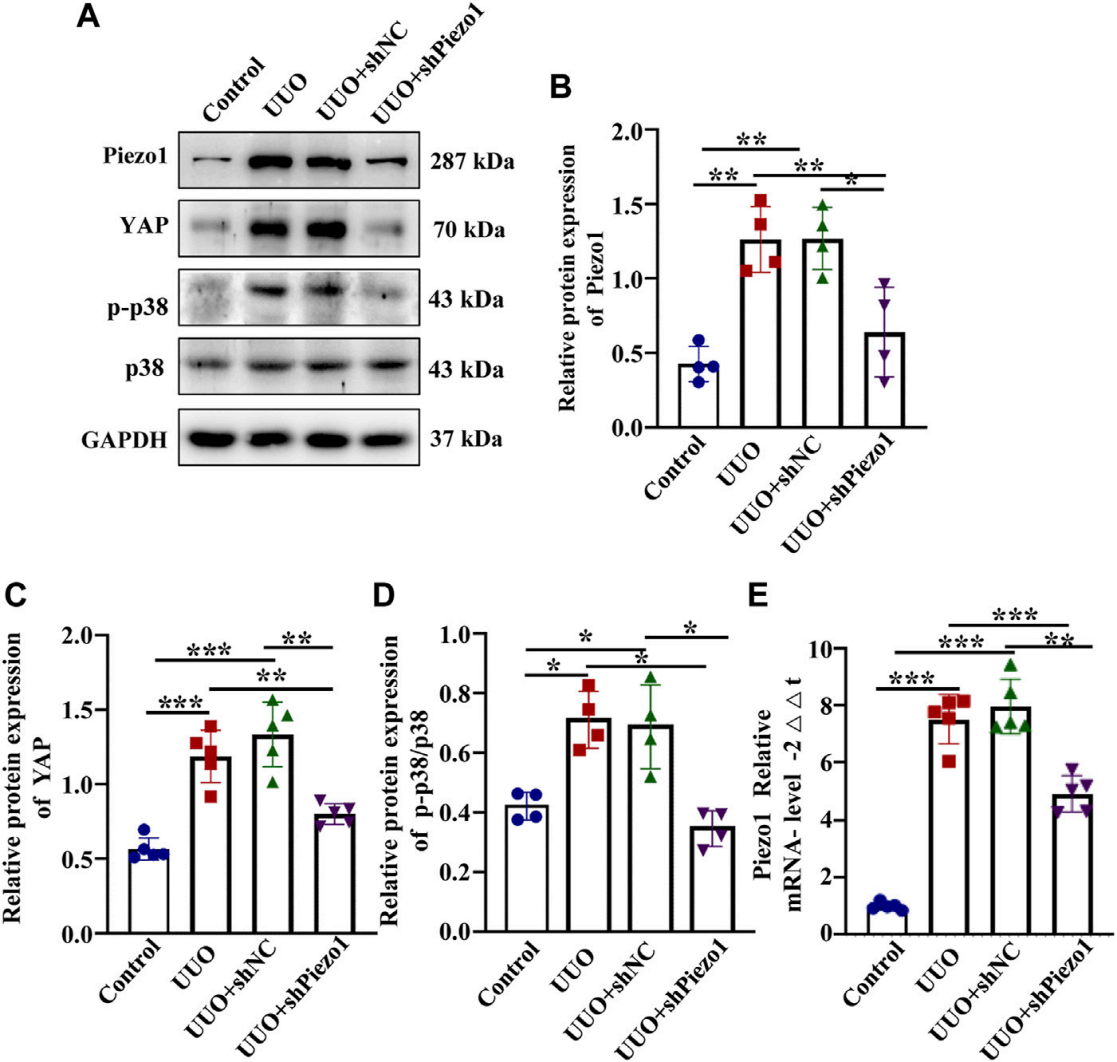
Piezo1 knockdown alleviates renal fibrosis through p38MAPK-YAP pathway. An animal model of renal fibrosis induced by UUO was established and a loss-of-function experiment was carried out by using shRNA of Piezo1 and shNC in UUO mice. **(A)** Representative western blot bands show the protein expression of Piezo1, p-p38, p38 and YAP in four different treatment groups. The bar graph shows the result of semi-quantitative measurement of Piezo1 **(B)**, p-p38/p38 **(C)** and YAP **(D)** (*n* = 4; *p < 0.05, **p < 0.01, ***p < 0.001). **(E)** Relative mRNA expression of Piezo1 in four different treatment groups. (*n* = 5, *p < 0.05, **p < 0.01, ***p < 0.001).

To verify that Piezo1 knockdown alleviate renal fibrosis through the p38MAPK-YAP pathway, we examined the expression of p-p38, p38 and YAP expression in four groups. As shown in Figures 10B,E, Piezo1 mRNA and protein level up-regulated significantly in comparison with the control, and they were downregulated after Piezo1 knockdown in UUO treated mice. Western blot results demonstrated that compared with the control, YAP protein leve and p-p38/p38 ratio were increased significantly in UUO group, but they were reduced significantly after Piezo1 sh RNA administrated in comparison with the UUO group (Figures 10C,D). Altogether, these results indicated that Piezo1 knockdown alleviated renal fibrosis induced by UUO through p38MAPK-YAP pathway.

## Discussion

It is generally accepted that changes in cellular mechanical microenvironment play an essential role in cell behavior and progression of disease (D'Urso and Kurniawan, 2020; Silver et al., 2021). In the present study, we investigated the mechanism of renal fibrosis from the perspective of mechanical force. Our results showed that ECM stiffness could promote excessive secretion of ECM by MCs through the Piezo1-p38MAPK-YAP signaling pathway, in addition, the Piezo1-p38MAPK-YAP signaling pathway have been verified play a role in the animal model of renal fibrosis induced by UUO and targeting mechanosensitive Piezo1 attenuates the progression of renal fibrosis.

YAP is the prime mediator of the Hippo pathway, and its expression and cellular distribution were reported to be mechanically regulated by ECM stiffness (Ghasemi et al., 2020; Zhang et al., 2020) In the present study, we found that the expression of YAP in kidney was elevated in response to UUO. In addition, the results demonstrated that YAP could be activated by enhanced ECM stiffness and translocate to the nucleus, moreover, the inhibition of YAP impeded CTGF expression and ECM secretion. Consistent with our results, Cheng *et.al*. reported that matrix stiffness can activate fibroblasts in a YAP-dependent manner (Liang et al., 2017). It was also reported that in fibroblasts, ECM stiffness mechanoactivates YAP, which promotes the production of profibrotic mediators and ECM proteins (Noguchi et al., 2018). Altogether, these results demonstrated that increased YAP-induced ECM production in MCs is mediated by enhanced ECM stiffness in renal fibrosis. However, how ECM stiffness regulates YAP expression and localization remains unknown.

Existing studies have shown that the entry of YAP into the nucleus is regulated by a variety of signaling pathway. However, as a key protein of Hippo signaling pathway, YAP has been reported participated in the Hippo signaling pathway in many studies and its mechanism is relatively clear (Yu et al., 2015). That is, the upstream regulatory element NF2 of Hippo signaling pathway can activate the core kinase chains Mst1/2 and LATS1/2 of Hippo pathway, and then regulate the activity and localization of YAP (Panciera et al., 2017). Bioinformatic analysis of protein-protein interactions in STRING v.10 provided a protein network associated with MAPK and YAP (Supplementary Figure S5). Moreover, cancer studies have demonstrated that enhanced matrix stiffness accelerates the migration of hepatocellular carcinoma cells via MAPK-YAP signaling (Qiu et al., 2020). However, it is not clear whether YAP is regulated by MAPK in renal fibrosis. Here, we established that p38 serves as an important link between ECM stiffness and YAP activation. p38, a member of the MAPK family, is a key regulator of proliferation, apoptosis and autophagy (Wang et al., 2017; Lee et al., 2020). Our results showed that p-p38, p-JNK, and p-ERK protein expression was significantly elevated in cells cultured on the stiff hydrogel compared with cells grown on the soft gel. However, only the p38 kinase inhibitor could prevent YAP activation induced by enhanced hydrogel stiffness. Guan *et al.* reported that the TEA domain (TEAD) family of DNA-binding transcription factors to which YAP binds to regulate gene expression, directly interact with p38 without scaffold proteins (Lin et al., 2018). Another report has demonstrated that p38, YAP, and TEAD are complexed together in cardiac fibroblasts (Bugg et al., 2020). The *in vivo* results also demonstrated that the p-p38 level was higher in the UUO model than in control mice. Previous studies have shown that the p-p38/p38 ratio is increased in renal fibrosis induced by UUO (Gu et al., 2019). In addition, mechanical force can stimulate the activation of p38MAPK to regulate cellular behavior (Bugg et al., 2020). It also has been demonstrated that p38 induces YAP nuclear translocation by inhibiting the components of Hippo pathway (Hsu et al., 2020). To investigate the effects of p38 inhibitor on Hippo components expression and the level of YAP phosphorylation. The results demonstrated that the p-MST1/MST1 and p-LATS/LATS increased significantly in cells treated with p38 inhibitor when compared to the control, and the expression of p-YAP also enhanced when added to p38 inhibitor (Supplementary Figure S6). Therefore, it is speculated that P38 may regulate Yap in two ways, the first one is that p38 can activate YAP by inhibiting Hippo pathway and the second is that p38 can directly regulate YAP without relying on other proteins. These results indicate that ECM stiffness serves upstream of the MAPK signaling axis and activates the phosphorylation of p38 MAPK, triggering YAP activation and a downstream cascade.

As p38MAPK is an intracellular molecule, it cannot directly sense surrounding mechanical signals. The membrane protein that senses mechanical signals and transduces these signals to p38MAPK to regulate renal fibrosis remains to be fully defined. Piezo1 has been reported to be an important sensor of various aspects of mechanotransduction (Wang et al., 2019). It is widely expressed in various tissues, including the kidney, and can be directly activated by mechanical forces acting on the cell membrane (Yoneda et al., 2019). Our results demonstrated that stiff substrate facilitates Piezo1 activation. Moreover, we also found that a significant increase in Piezo1 expression in renal fibrosis induced by UUO. *In vitro* results showed that Piezo1 silencing reduced ECM expression and YAP nucleation. In addition, YAP expression and ECM secretion dramatically increased after administrated the Yoda-1, whereas they were reduced significantly in cells treated with Yoda-1 and SB203580 when compared with cells applied for Yoda-1 alone. These results further demonstrate that p38MAPK involved in the regulation of YAP and ECM levels by Piezo1. Moreover, *in vivo* experiment showed that Piezo1 knockdown could alleviate renal fibrosis and renal function, and which the process mediated by the p38MAPK-YAP pathway. Piezo proteins are subunits of calcium ion-permeable nonselective cation channels that respond to mechanical force (Liu et al., 2020). When Piezo1 activated by mechanical force, it causes a massive influx of calcium ions into the cell. It has been reported that the increase of calcium in cells is related with p38 MAPK activation (Zhou et al., 2021). Turner *et al.* reported that p38 is activated downstream of Piezo1-mediated Ca^2+^ entry, increasing IL-6 secretion (Blythe et al., 2019). Also, in bone homeostasis, Piezo1 has been reported that induce YAP-dependent expression of several ECM such as type II and IX collagens (Wang et al., 2020). However, it has been reported that Piezo1 could act upstream of YAP in oral squamous cell carcinoma (Hasegawa et al., 2021), We speculate that the hierarchical relationship between PIEZO1 and YAP may be dependent on this cell context, or that there is a positive feedback regulation between PIEZO1 and YAP.

In conclusion, elevated ECM stiffness could activate Piezo1, which increased YAP activity through the p38MAPK. This increased YAP protein promotes ECM secretion, thereby contributing to the progression of renal fibrosis. Moreover, the progression of renal fibrosis can be alleviated by intervening Piezo1. These results highlight the importance of Piezo1 and the potential utility of Piezo1 as a biomarker and therapeutic target of renal fibrosis.

## Data Availability

The original contributions presented in the study are included in the article/Supplementary Material, further inquiries can be directed to the corresponding author.
