# Supplementary material for: Targeting Mechanosensitive Piezo1 Alleviated Renal Fibrosis Through p38MAPK-YAP Pathway
Source: Front Cell Dev Biol. 2021 Nov 5;9:741060. doi: 10.3389/fcell.2021.741060 (PMC8602364; doi:10.3389/fcell.2021.741060)
Supplement: Supplementary file 1 [file DataSheet1.docx]

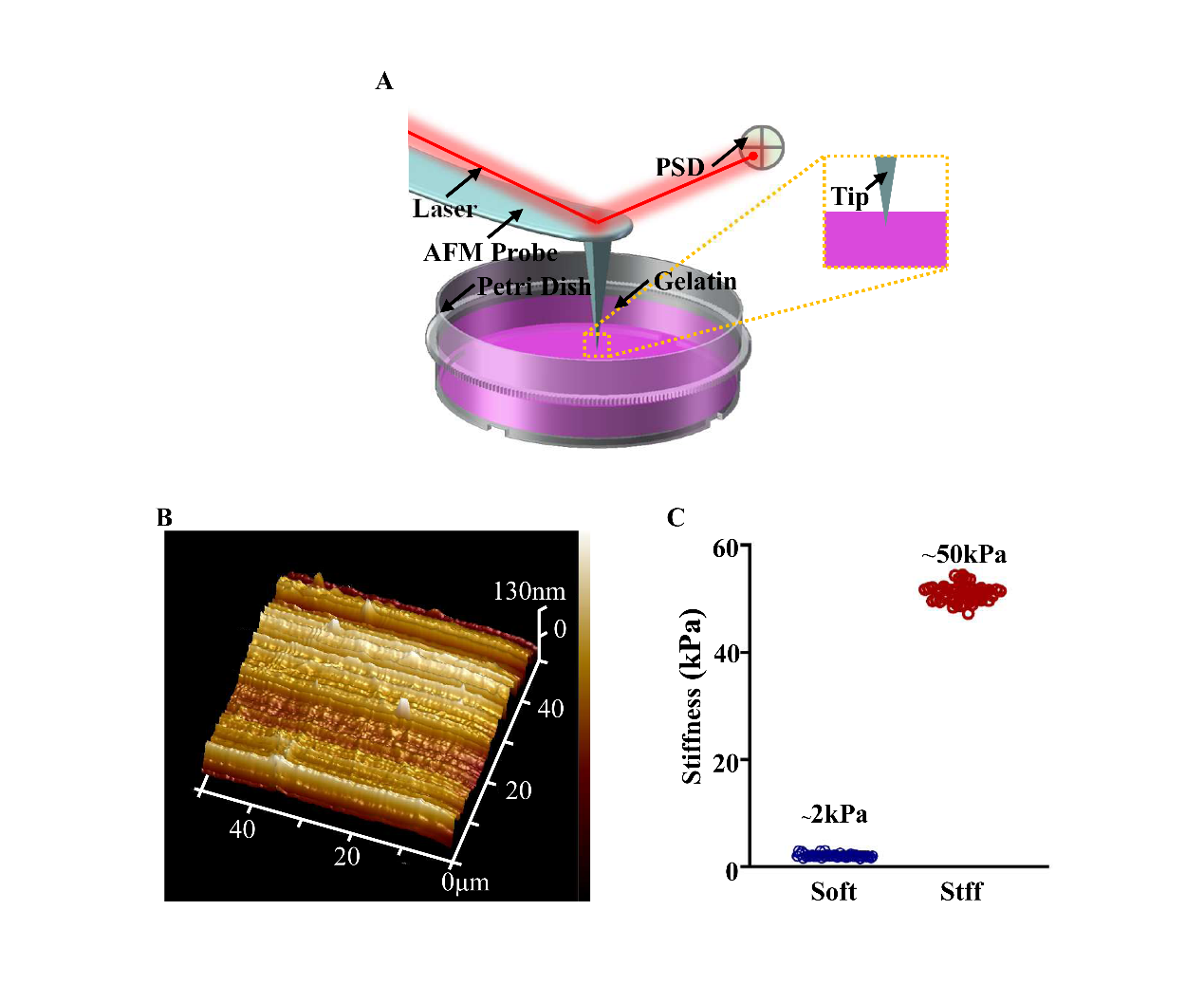


**Figure S1. Substrate stiffness measurements of the hydrogels.** (A) Diagram of the working principle of AFM. (B) Schematic diagram showing the hydrogel surface flatness. (C) Results of hydrogel stiffness measurements.


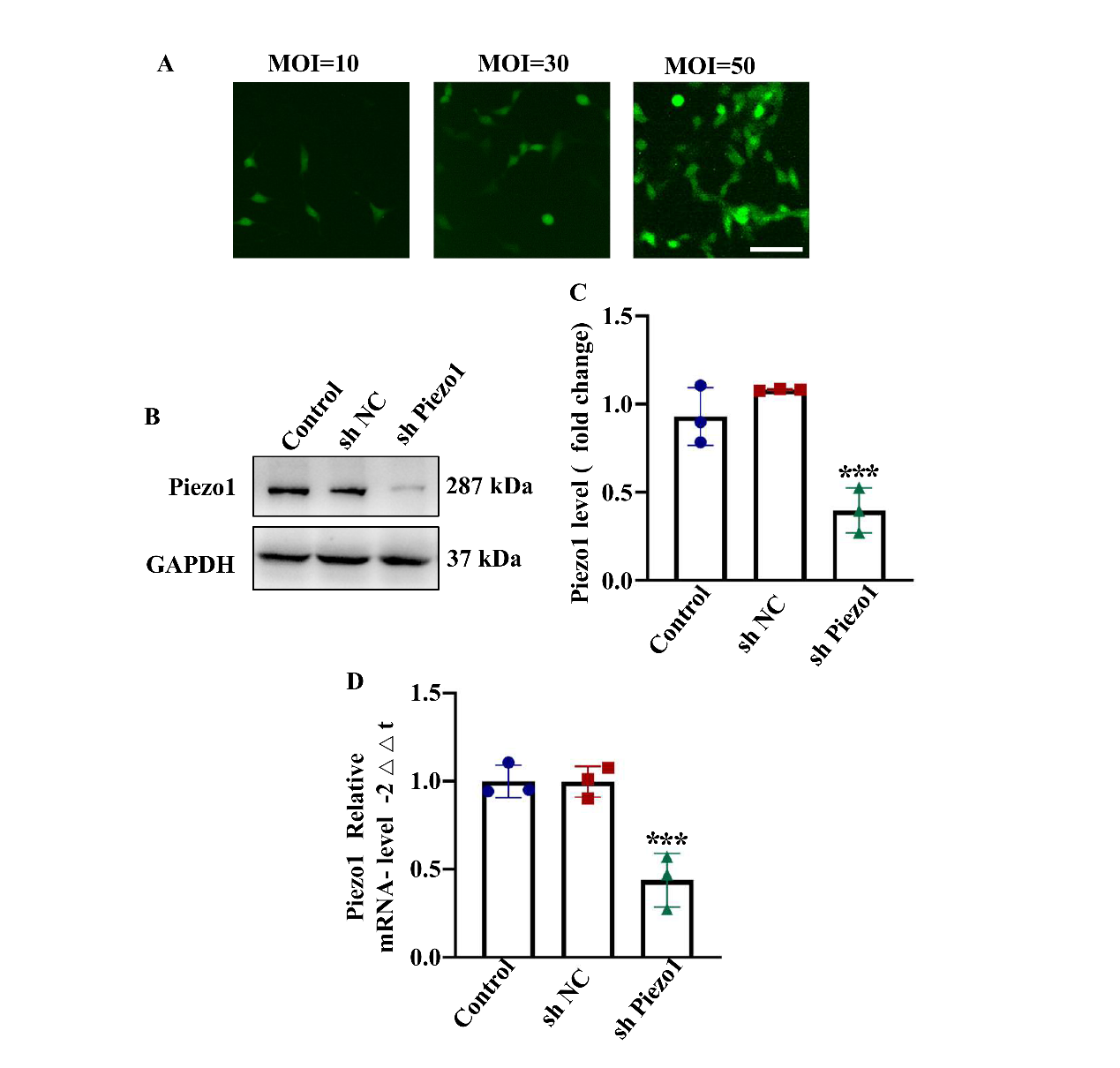


**Figure S2. Transfection efficiency of lentivirus with Piezo1 shRNA sequence.** (A) Multiplicity of Infection (MOI) gradient of Pizon1 lentivirus infecting MCs at 24 hours post infection, scale bar indicates 25 μm. (B) Transfection efficiency was tested by measuring Piezo1 by immunoblot assay (n=3, *, *P*<0.05; **, *P*<0.01). (**C**) Transfection efficiency was tested by measuring Piezo1 by PCR (n=3, *p < 0.05, **p < 0.01, ***p < 0.001).


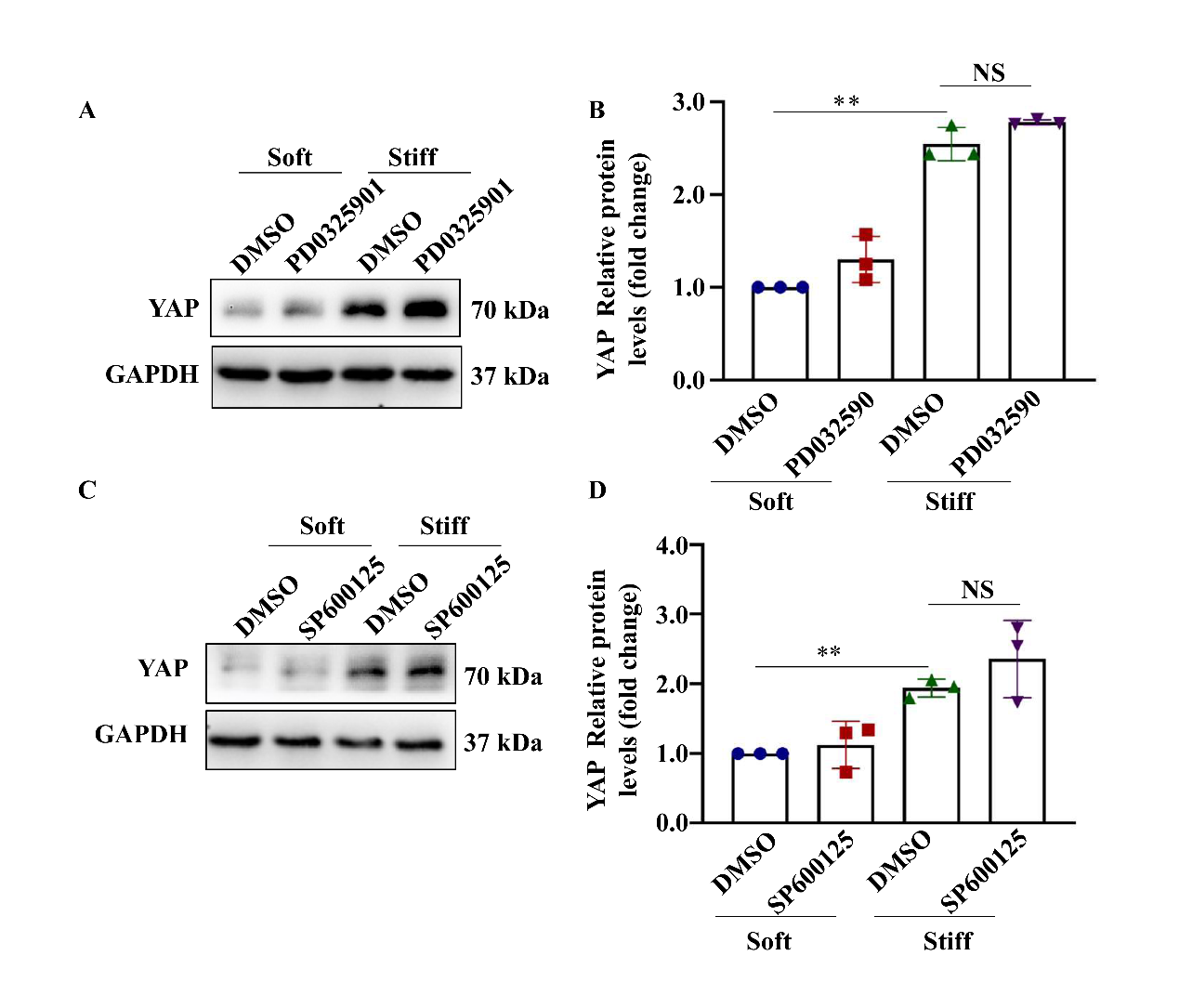


**Figure S3. SP600125 and PD0325901 treatment did not alter the expression of YAP.** MCs were treated with vehicle or inhibitors on different stiffness GelMA hydrogels. Immunoblot analysis of YAP in cells treated with PD032590(A) and SP600125(C). (B-D) The quantification of YAP after corresponding inhibitor treatment. Error bars indicate mean ± SD, n=3 (*p < 0.05, **p < 0.01, ***p < 0.001), NS, not significant.


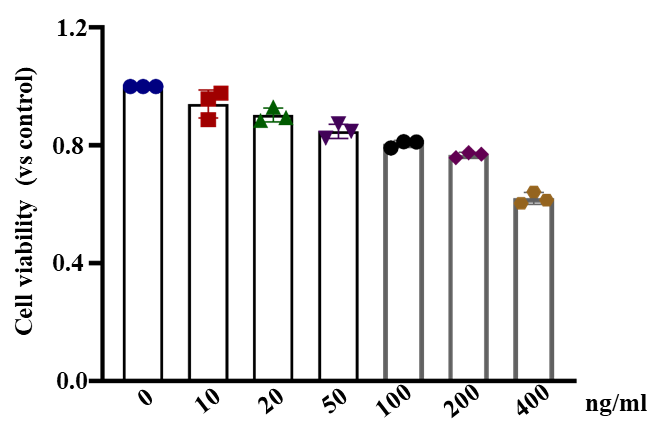


**Figure S4. Effects of Super-TDU on cell viability of MCs.** MCs were exposed to various concentrations Super-TDU for 24 h, and cell viability was measured by CCK-8 kit. Data are expressed as the three independent experiments. Each assay is performed in triple.


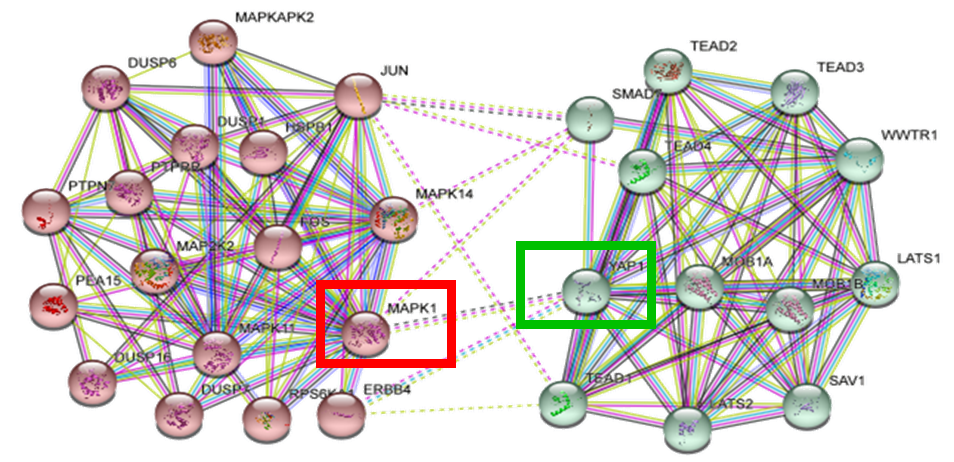


**Figure S5.** Bioinformatic analysis of protein-protein interaction networks in STRING v.10 shows a network associated with MAPK and YAP.


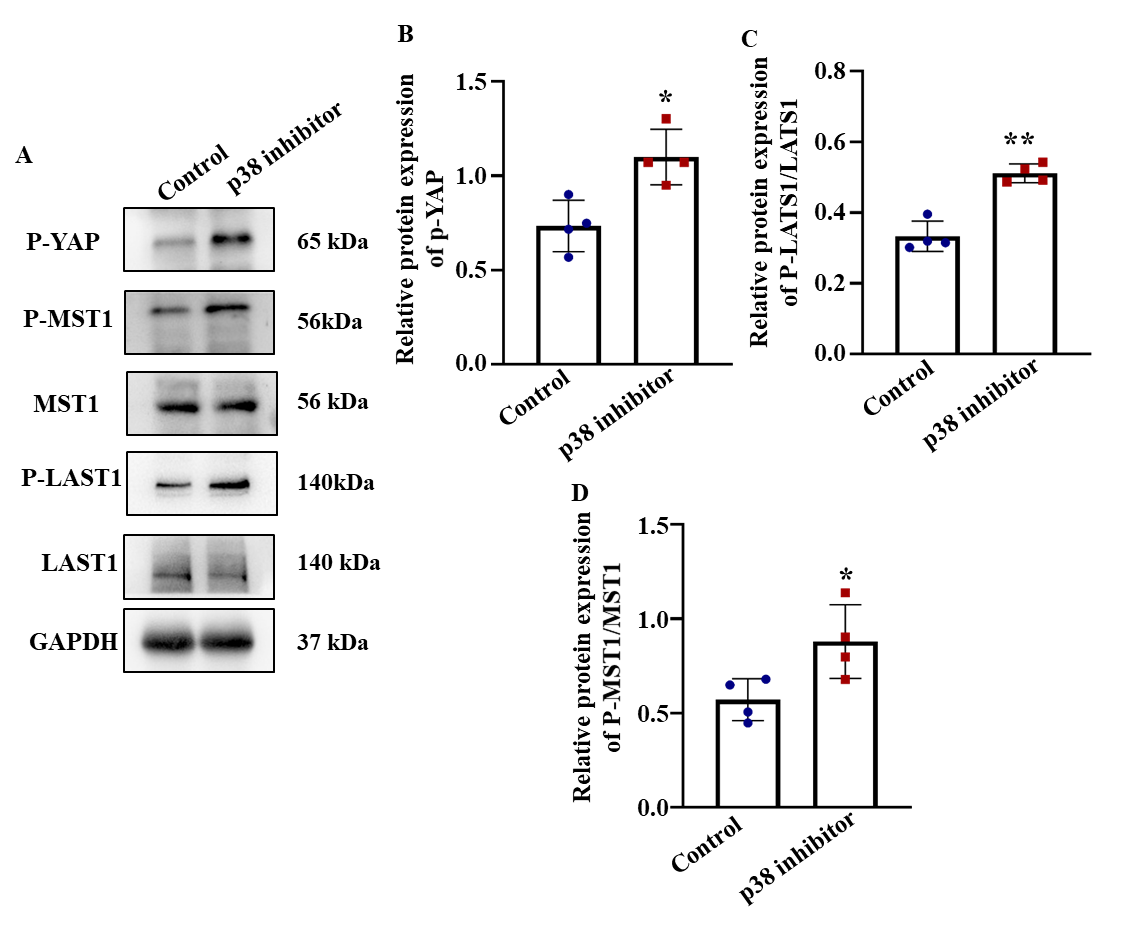
**Figure S6.** **p38 inhibitor activated the Hippo pathway and then increased p-YAP expression.** The p38 inhibitor was added to the cells cultured on stiff gel for 24h, the Hippo components consists of p-MST1, MST1, p-LATS1 and LATS1 were measured. (A). Representative Western blot bands showing p-YAP, p-MST1, MST1, p-LAST1 and LAST1 expression in each group. Semiquantitative measurement of YAP (B), p-MST1/ MST1 and p-LAST1/LAST1 (mean ± SD, n =4; *p < 0.05, **p < 0.01, ***p < 0.001).
